# Ovarian Large Cell Neuroendocrine Carcinoma Associated with Serous Carcinoma: Correlation of Pathology with MR Imaging

**DOI:** 10.2463/mrms.ci.2016-0150

**Published:** 2017-05-31

**Authors:** Mayumi Takeuchi, Kenji Matsuzaki, Koichi Tsuneyama, Masato Nishimura, Eri Takiguchi, Masafumi Harada

**Affiliations:** 1 Department of Radiology, Tokushima University, 3-18-15 Kuramoto-cho, Tokushima, Tokushima 770-8503, Japan; 2 Department of Pathology and Laboratory Medicine, Tokushima University, Tokushima, Japan; 3 Department of Obstetrics and Gynecology, Tokushima University, Tokushima, Japan; 4 Department of Radiological Technology, Tokushima Bunri University, Kagawa, Japan

**Keywords:** large cell neuroendocrine carcinoma, serous carcinoma, ovary, magnetic resonance imaging

A 58-year-old woman with progressive lower abdominal fullness and pollakiuria was referred to our hospital. A well-demarcated right adnexal, solid-dominant mass of 14 cm in diameter showed heterogeneous signal intensity on T_2_-weighted images (T_2_WI) (Fig. 1a and b) and diffusion-weighted imaging (DWI) (Fig. 1c), and heterogeneous contrast-enhancement on post-contrast T_1_-weighted images (T_1_WI) (Fig. 1d) due to the presence of hemorrhage, necrosis, and cystic components. Multilocular cystic components with thickened septa and nodules exhibiting high intensity on DWI with low apparent diffusion coefficient (ADC) and intense contrast-enhancement were also observed in the posterior portion of the mass. A multilocular cystic mass of 6 cm in diameter similar to the multilocular cystic components of the right mass was revealed in the left adnexal region. Prominent intra-tumoral hemorrhage as massive signal voids on susceptibility-weighted imaging (Fig. 1e), and hypervascularity and plateau after rapid increase dynamic curve pattern on dynamic contrast-enhanced (DCE)-MRI were suggestive for its malignant nature. Bilateral ovarian cancers of different histology were suspected based on the magnetic resonance imaging (MRI) findings. The patient was diagnosed with stage II disease at surgery. Histological examination of the right mass (Fig. 2a) revealed a combination of large cell neuroendocrine carcinoma (LCNEC) and high-grade serous carcinoma (HGSC) (Fig. 2b, c), whereas the left mass was diagnosed as HGSC. LCNEC component was considered as arising from HGSC.

LCNEC is a rare, aggressive poorly-differentiated neuroendocrine tumor usually associated with other epithelial neoplasms as its origin, and pure LCNEC is extremely rare.^1,2^ Associated ovarian cancer is mainly mucinous carcinoma, and endometrioid or serous carcinoma is less common.^1,2^ That may be because mucinous carcinoma tends to show neuroendocrine differentiation than other subtypes. LCNEC and associated carcinoma have similar genomic profiles suggesting monoclonality in origin, and LCNEC has additional genetic abnormalities in comparison with associated carcinoma suggesting that dedifferentiation of associated carcinoma may cause LCNEC.^2^ Taube experienced a case of HGSC with metastasis of LCNEC component^3^ and analyzed 178 HGSC, and synaptophysin expression was found in 6.7% of cases suggesting neuroendocrine differentiation. Synaptophysin expression (>20% of positive cells) was revealed as significant prognostic factor in multivariate analysis.^3^ This result suggested that a minor neuroendocrine differentiation might be more frequent in HGSC than suspected by morphology.

Because LCNEC is usually associated with other epithelial neoplasms, an aggressive adnexal mass with co-existing tumor exhibiting different morphological appearance on MRI may suggest this rare malignancy.

## Figures and Tables

**Fig 1.**
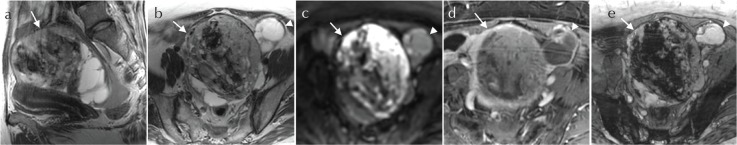
Right adnexal solid-dominant mass with multilocular cystic components (arrow) and left multilocular cystic mass (arrowhead) on T_2_-weighted images (T_2_WI) (repetition time [TR]/echo time [TE]:5000/97-99 ms) (**a**, **b**), diffusion weighted image (DWI) (4000/51 ms, b = 800 sec/mm^2^) (**c**), post-contrast T_1_-weighted images (T_1_WI) with fat-suppression (4.0/1.7 ms) (**d**), and susceptibility-weighted imaging (T_2_ star-weighted angiography [SWAN]) (42/27 ms) (**e**).

**Fig 2.**
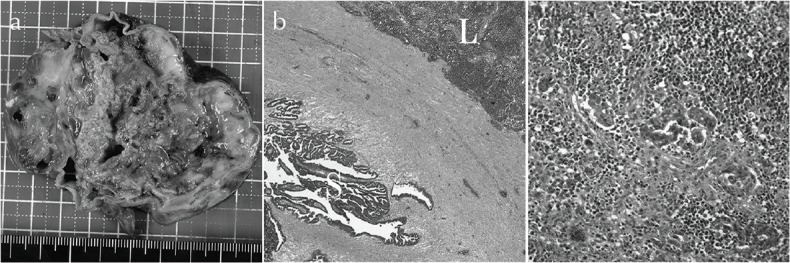
Massive hemorrhage and necrosis are observed on the cut surface of the resected right adnexal mass (**a**). Photomicrographs (hematoxylin and eosin) show a combination of large cell neuroendocrine carcinoma (LCNEC) (L), which is consisted of solid islands of large cells with large nuclei exhibiting positive for synaptophysin and CD56, and high-grade serous carcinoma (HGSC) (S). LCNEC and HGSC are present in close proximity (**b**) and are mixed in part (**c**).
